# *Polyura inopinatus* Röber, 1940; a remarkable butterfly mystery resolved

**DOI:** 10.3897/zookeys.774.26458

**Published:** 2018-07-12

**Authors:** Chris J. Müller, W. John Tennent

**Affiliations:** 1 Research Associate, Australian Museum, 6 College Street, Sydney, NSW 2010, Australia; 2 Scientific Associate, Department of Life Sciences, the Natural History Museum, London SW7 5BD, UK; 3 Honorary Associate, Oxford Museum of Natural History, Parks Road, Oxford OX1 3PW, England; 4 Address for correspondence: PO Box 3228, Dural, NSW 2158, Australia

**Keywords:** Taxonomy, Lepidoptera, Nymphalidae, Charaxinae, *Charaxes*, *Polyura*, neotype, Bismarck Archipelago, Sulawesi

## Abstract

The most distinctive species of *Polyura*, *P. inopinatus*, described from a single specimen said to be from North Sulawesi, Indonesia, has been a great mystery since it was first described by Röber, in 1940. The holotype, originally illustrated in monochrome in the journal *Deutsche Entomologische Zeitschrift, Iris*, was lost very soon after it was described, almost certainly destroyed during allied bombing of Dresden in the 1940s. No other specimen was known for almost eight decades. We suggest that the type locality (Sulawesi) is incorrect and that the holotype was more likely to have been collected in the Baining Mountains, East New Britain Province, Papua New Guinea. We report the recent discovery of several male *P. inopinatus* from West New Britain Province, and describe and illustrate specimens. A neotype is designated.

## Introduction

### Background

Johannes Karl Max Röber (known as Julius) (1861–1942) was a Dresden entomologist. He became a prolific author from the age of 25, describing many new butterfly taxa between 1886 and 1940. His final paper concerning the Lepidoptera was a detailed (annotated) checklist of the 406 butterfly species known to occur on Celebes (now Sulawesi), Indonesia, which included description of three new taxa: *Appias melania kalisi* (Pieridae) (Röber, 1940: 93); *Charaxes inopinatus* (Nymphalidae) (Röber, 1940: 102) and *Celaenorrhinus chamunda subconcolor* (Hesperiidae) (Röber, 1940: 113).

There is some divergence of opinion regarding the publication date of Röber’s paper. Authors of the Sulawesi checklist (Vane-Wright and de Jong 2003) and the most recent treatment of *Polyura* (Turlin 2017 a, b, c), used the year 1939 for the name *inopinatus*, but we note that Vane-Wright and Ackery (2003: 27, 187) used 1940 for *subconcolor* (Vane-Wright and de Jong 2003: 61). Yata et al. (2010: 796, supporting information, 13) subsequently used the year 1940 for the name *kalisi*. The journal itself is clearly dated 1939, and was the final issue of that year. However, it was actually published on the 10^th^ of April 1940 (Matthias Nuss, in litt., 2018).

**Figures 1, 2.**
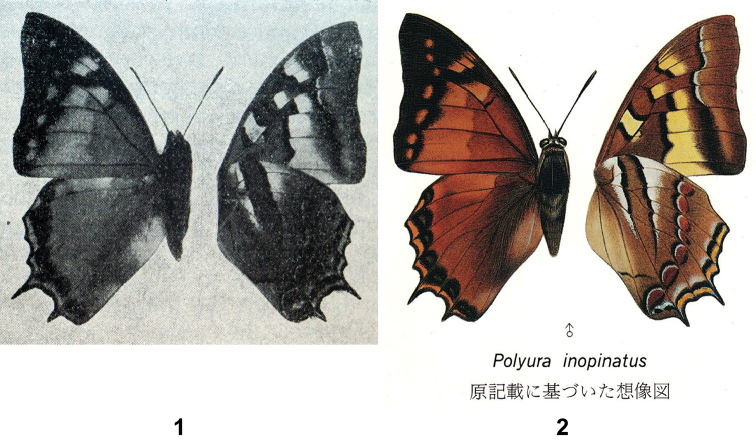
*Polyura inopinatus* holotype: **1** as illustrated by Röber (1940) **2** as prepared by Toshitsugu Endo and reproduced in colour in Tsukada (1991).

Of the three new taxa described in his paper, *inopinatus* (inopinatus means ‘unexpected’ in Latin) was clearly – and rightly – regarded by Röber as the most important / impressive, since it was the only butterfly to be illustrated in his paper (Fig. 1). Röber’s checklist was primarily based on the previous work of Ludwig Martin (1858–1929) (see references), who lived on Sulawesi between 1914 and 1929, and collected butterflies there for some of this period. However, the solitary specimen of *inopinatus* was not from Martin, but was said to have been “captured in North Celebes (Tondano) and comes from the stores of Dr. Staudinger and A. Bang-Haas in Dresden-Blasewitz. It is probable that this new species has its home in the mountains, and in the lowlands only specimens blown by the wind are encountered. As yet no mountain of the island of Celebes is sufficiently explored, many surprises are to be expected in the future” (Röber 1940: 104 [translation]).

An English language translation of relevant sections from Röber’s rather long original German language description, is provided here:

“*inopinatus* sp. n. is the latest and most remarkable discovery of the island of Celebes. This new species has no nearer kinship in the whole genus, and only a few resemblances on the underside of the wings with *cognatus*, also from Sulawesi, which is also without a close relative. The upper side is light reddish-brown with black markings…”

At the middle cell is a large black spot, which connects with the black margin; The anterior is approximately 1mm wide, black and the discocellulars are narrowly black sculpted, the remaining ribs are narrowly black.

Posterior wing parts are wider and diffuse yellowish.

The outside edge is monochrome brownish; the proximal wing part is brown; in the centre of the cell there is a large yellowish-yellow spot, on both sides a broad black spot, the cell is closed by a black stripe. The hind wings are grey-brown, and in the centre there is a darker band, about 3 mm wide, bounded by silvery white; This white band widens distally to an oblong triangular patch which runs into the anterior margin; distally 7 reddish-brown spots, partly semicircular, which are proximally bluish-silver-white, and then narrowly black.

The antennae are black, the palps are black, the abdomen is yellow, the thorax is brighter and the legs are black, yellowish below. Wing span 50 mm.”

### Where is the *inopinatus* holotype?

The unique specimen from which Röber described *inopinatus* is lost. Turlin’s rather subjective critical assessment (Turlin 2017b: 15) that the specimen was lost as a result of “the terrible bombings of 13/14 February 1945”, whilst fundamentally accurate, is ultimately unhelpful. The true story is more complex. Bombing of Dresden in February 1945 has been offered as the explanation for the wholesale destruction of materials including natural history collections at what is now the Senckenberg Museum für Tierkunde in Dresden. This has provided anecdotes – for example, the only specimens to escape the bombing were those out on loan at the time – that are clearly nonsense.

An objective, interesting and factual account of the history of the collections before and during the war was presented by Robert Reichert (1954, 1956), a member of the museum staff who felt moved to make an historical record covering major changes from the period 1937 until almost a decade after the end of the war, when his papers were published.

The Nationalsozialistische Deutsche Arbeiterpartei (NSDAP – usually referred to in English as the Nazi Party) was formed in 1920, and rose to prominence in the following decades, before being declared illegal in 1945, immediately following the end of the Second World War. This was a dark period in European history, but prevalent attitudes of those times may also be relevant to our story of what may have happened to the type (holotype) of *Polyura inopinatus* described by Röber in 1940 at the beginning of the war.

Dresden was the most important cultural centre in Saxony, with the world famous Semper Oper House and a number of other important and magnificent buildings which included the Zwinger building – part of the city of Dresden when it was constructed in the early 18^th^ century – which now houses the Old Masters Picture Gallery, the Dresden (Meissen) Porcelain Collection, and a museum of Mathematical and Physical instruments.

Until the 1930s, the perimeter buildings (the Zwinger is in effect a series of buildings linked by high walls, enclosing a large open area) housed ‘museums’ (collections) including ethnology, zoology and mineralogy. Under the influence of the Nazi regime, the museums of ethnology and zoology were re-organised to the “Staatliches Museum Tierkunde, Völkerkunde und Rassenkunde” (State Museum of Zoology, Ethnology and [Race Theory]) and the Nazis ordered the movement of the contents of this Museum from the Zwinger to another building in the Ostra-Alle 15, which was completed by the year 1937. In 1939, some valuable books and type specimens of birds were secured there.

With a likelihood of allied bombing as early as 1940, movement of museum objects for safe keeping was ordered on 15^th^ February 1940 to 16 different ‘safe’ localities in castles and other major buildings throughout the State of Saxony – although some of these buildings were also severely damaged during the war, with resultant loss of museum material. The 7^th^ of October 1944 was a bleak day for the Dresden Museum, where much of the entomology collections remained. The building suffered a direct hit around mid-day, causing a fire in the museum that burned for 12 hours. The bulk of the Entomology Department was completely destroyed, including the larger part of the butterflies, the moths, all Hymenoptera, one cabinet of Coleoptera and the important collection of Heinrich Wilhelm Calberla (1839–1916).

Most of what remained of the zoology collections in Dresden was destroyed by intensive bombing on 13^th^-15^th^ February 1945, which caused massive destruction of the City Centre. Most of the Museum documentary records were also destroyed as a result of the October 1944 and February 1945 bombings.

Immediately following the war, problems continued to beset the surviving residue of the entomology collections. Staff members visited all the ‘safe’ localities in the summer of 1945, to find extensive local damage. At one locality 17 insect cabinets were found to have been destroyed, at another locality fire had destroyed a large proportion of the library. Return of surviving collections to Dresden following the war was, predictably, problematic since most of the buildings suitable to house collections in the centre of Dresden were either destroyed or rendered uninhabitable. Space was at a premium, and much of the returning material remained packed in boxes for many years.

In summary, a significant volume of entomological material, including we presume the unique specimen of *inopinatus* perished as a direct influence of war. Concurrent loss of museum records leaves us with no explanation of what actually happened to the specimen, and the truth cannot now be established. But what can be presumed, with a high degree of certainty, is that any specimen that might be expected to have been in Dresden and which is now neither in the Senckenberg Museum für Tierkunde (Museum of Zoology) in Dresden nor in the Museum für Naturkunde (Museum of Natural History) in Berlin, was destroyed during these raids.

### Subsequent references to *inopinatus*

Subsequent published history, comment and opinion regarding *inopinatus* is sufficiently fascinating to be worth recording in detail. The butterfly was described by Röber in the genus *Charaxes* but note that we continue to use *Polyura* in the sense of Smiles (1982) for the group of Indo-Australian Charaxini with an open hindwing discal cell.

There seems to have been no further mention of *inopinatus* until the spectacularly illustrated work of Tsukada (1991). Smiles (1982) revised *Polyura*, but overlooked *inopinatus*; D’Abrera (1985) – undoubtedly using Smiles as a primary reference – also neglected to acknowledge its existence.

### Tsukada

Tsukada’s series of *Butterflies of the South East Asian islands* is a five volume monographic work which was intended to include all the butterflies of the region, although the lycaenids and hesperiids were never published. But *Polyura inopinatus* was presented with a magnificent colour illustration (Tsukada 1991: 236) (Fig. 2) with the legend/text “*Polyura inopinatus*, imaginary picture, drawn based upon its original description [translation]” (but see below). The drawing / painting was made by Toshitsugu Endo (Tsukada 1991: 518) and rather artistically superimposed over the German text from Röber’s description.

### Vane-Wright and de Jong

In a comprehensive checklist of Sulawesi Region butterflies, Vane-Wright and de Jong (2003: 27) noted “The endemic species that Smiles unfortunately overlooked, *Polyura inopinatus*, is very distinctive. It is known only from the unique holotype, described from northern Sulawesi in 1939 [sic], and its place within Smiles’ scheme has not been determined. *P. inopinatus* was not encountered during Project Wallace” (see below for discussion on ‘Project Wallace’) and later (Vane-Wright and de Jong 2003: 187) “Described by Röber (1940) from a single male from Tondano, this distinctive taxon was overlooked by Smiles (1982). Tsukada (1991: 236) reproduces the original colour illustration; so far as we are aware, no further material of this beautiful species has come to light”.

Reference to “the original colour illustration” is erroneous. Röber’s paper was published at the beginning of the Second World War, when it was difficult to obtain issues of German-published journals. The volume in the library of the Natural History Museum in London is a photocopy, and it might reasonably be assumed that the original was actually in colour. However, the second author, during a visit to the Senckenberg Museum für Tierkunde in Dresden in November 2017, examined an original copy of the journal, and found the illustration to be monochrome.

We know now that Tsukada’s colour illustration was prepared by Toshitsugu Endo from a combination of a Japanese translation of Röber’s original German description and the monochrome illustration. It is noted that the size of the specimen – in reality very small indeed in comparison to other Indo-Australian species of *Polyura* or *Charaxes* – was apparently overlooked. Although Röber (1940: 104) did provide a wingspan (“Flügelspannweite 50 mm”), Tsukada’s illustration (Tsukada 1991: 236, between plates 202, 203) depicted the upperside and underside ‘halved’, each half of which was *ca* 30 mm; a space between the two halves gave the impression of a butterfly with a span of approximately 70 mm. Tsukada noted ‘upper side *Charaxes*-like, under side *Polyura*-like, looking like a monster” (Tsukada 1991: 518 [translation]), which we take to have meant that it seemed aberrant in some way.

### Toussaint, Turlin and a ‘hybrid origin’

More recently, Toussaint et al. (2015) presented a phylogeny of *Polyura* sensu stricto (Indo-Australian region) that included all known taxa with the exception of *inopinatus*, for the obvious reason that no specimen was available to the authors, although Tsukada’s coloured illustration was included (Toussaint et al. 2015: 198), without acknowledging the source. It was accompanied by the comment (Toussaint et al. 2015: 197) “*P. inopinatus* is highlighted in a red rectangle to indicate its likely extinction *in natura*. A drawing of this species is presented since the monotype was destroyed during World War II)”. The possibility of a hybrid origin for *inopinatus* was suggested: “... Our dataset includes 205 specimens representing all described species except for the dubious Sulawesi endemic *P. inopinatus* which is known only from the lost holotype and may be a hybrid” (Toussaint et al. 2015: 195).

A modern treatment of *Polyura* was presented by Turlin (2017a, b, c). He said of *inopinatus*: “...the single male specimen, reputed to have come from Tondano, near Manado, was deposited in Dresden Museum collection just before the beginning of the 2^nd^ world war. It unfortunately disappeared at that time, presumably destroyed during the terrible bombings of 13 and 14 February 1945, which destroyed most of Dresden. Only the original description, with a black and white photo, remains as proof of its existence ... Tsukada managed to publish a colour painting from the original description text ...” (Turlin 2017a: 21). As will be seen from our previous section, above, the specimen was almost certainly destroyed during the World War II allied bombing of Dresden, although this was not as straightforward as Turlin suggested. Turlin went on to say (Turlin 2017b: 15) that he considered a Sulawesi origin may be erroneous and that *inopinatus* “... could have come from another island, maybe a very small one [presumably near Sulawesi], which was visited only once and never again since the discovery and still remains unidentified ... the pity is that the only specimen is lost forever. This case will remain a mystery for a long time!”.

Potential for a ‘hybrid origin’, raised by Toussaint et al. (2015: Turlin was a co-author of that paper) and Turlin (2017b: 15), as an explanation for a lack of available specimens, was unconvincing. Such a scenario would require the presence, in the same place, of suitable parents and an expectation that a hybrid offspring would display characters of both. Such potential parents were and are not present on Sulawesi – or anywhere else – and although a hybrid possibility was investigated in some detail by Turlin (2017b: 15), it is not pursued here, in part because there is no evidence whatever for a hybrid origin, but also because of the recent discovery of the species in Papua New Guinea.

## Materials and methods

Adult specimens were photographed using a Nikon D300s Digital SLR Camera with a Nikon AF-S VR Micro-Nikkor 105mm f/2.8G IFED Macro lens and Nikon R1C1 Close-up Kit Flashes Speedlights. Male genitalia were photographed in glycerol using the fore-mentioned camera body adapted to a Meiji Techno EMZ-5TR-P-FOI Trinocular Stereozoom Microscope, with OPTEK FL95E Fibreoptic Illuminator and twin arm optical fibre, after being extracted following maceration of abdomens in 10% KOH at room temperature for 36 hours. Individual images were taken with the remote acquisition software DIYPhotoBits Camera Control 5.2. Sliced genitalia photographs were stacked and concatenated using the software Helicon Focus 6.0 and edited in Adobe Photoshop CS6. Image plates were designed in Adobe InDesign CS6. Genitalia were stored in small glycerol-filled vials pinned beneath the specimen.

## Results

### The re-discovery of *Polyura inopinatus*

During early September, 2015, whilst carrying out unrelated research into the lycaenid genus *Hypochrysops* on West New Britain, in the Bismarck Archipelago, the first author glimpsed what appeared initially to be a particularly small species of black and orange *Doleschallia* butterfly (Nymphalidae) alighting briefly on an isolated branch some 20 metres above the ground. Some days later, a second butterfly observed in the same place was examined through binoculars; it appeared to have an under surface similar in appearance to that of a species of *Polyura* and an upper surface that appeared to be largely orange. With some difficulty, over a period of several days, five male specimens were collected.

It was clear from the outset that, despite its presence some 3,000 kilometres to the east of its published type locality on North Sulawesi, this was *Polyura inopinatus*. Specimens from West New Britain are very similar in shape and maculation to the specimen illustrated in Röber (1940) (see figs). The diminutive (for a *Polyura*) wing span (50 mm) given by Röber is also accurate when compared to our specimens. There can be little doubt that the specimens from New Britain are the same as that supposedly from Sulawesi, and that the original claim for a Sulawesi origin is at best highly dubious. The female was not seen; males of *P. inopinatus* were observed to be very high flying, establishing territories in the canopy close to the summit of a tall volcanic hill. The locality was completely destroyed by fire towards the end of the severe drought (El Niño) of July–December 2015.

**Figures 3–8.**
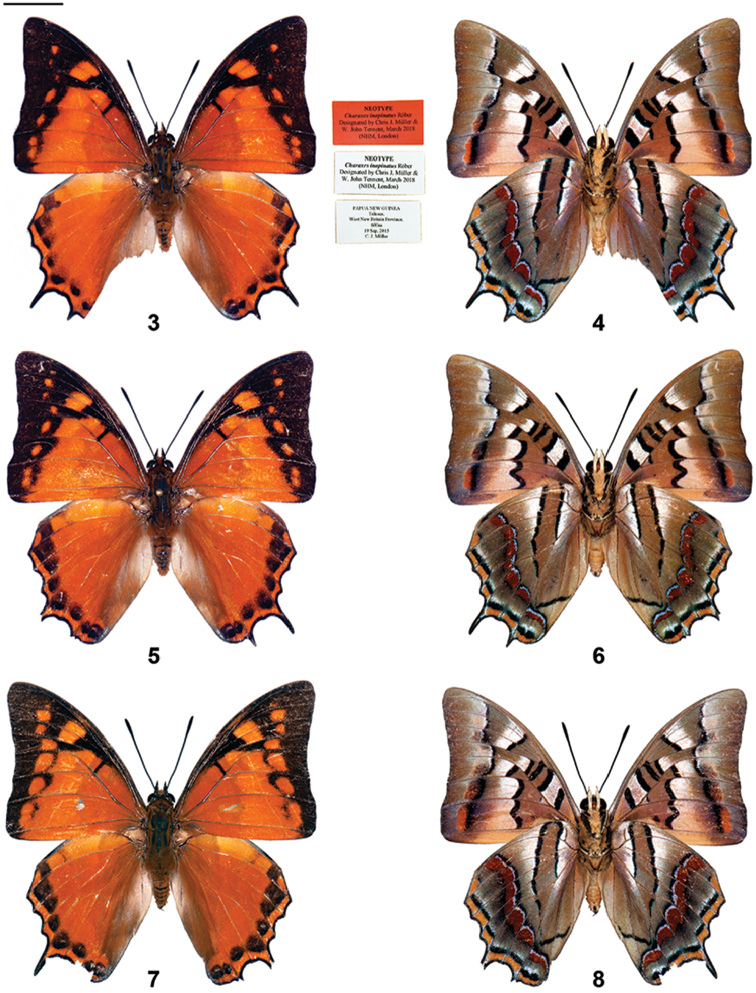
*Polyura inopinatus* males, Talasea (PNG) (odd numbers upperside, even numbers underside): **3, 4** Neotype male, with labels **5, 6** additional male **7, 8** additional male. Scale bar = 10 mm.

The second author has examined every nymphalid drawer in the Senckenberg Museum für Tierkunde in Dresden and both authors have examined the nymphalid drawers of the Staudinger and general collections in the Museum für Naturkunde, Berlin. Others have also searched for the specimen in previous years. There is no doubt that the holotype of *P. inopinatus* is lost, almost certainly associated with the bombing of Dresden during World War II. As will be clear from our reproduction of Röber’s monochrome picture, Tsukada’s colour representation, and fresh specimens, there is equally no doubt (cf Figs 3–8) that the butterflies found on West New Britain represent *inopinatus*.

The International Code of Zoological Nomenclature (The Code) requires that a neotype designation should not be made for “curatorial convenience”, but that factors including taxonomic status or the type locality of a nominal taxon should be considered. In addition to fascinating historical aspects of the “re-discovery” of *inopinatus*, we are confident that the unusual and unique circumstances we have outlined clearly warrant designation of a neotype. We also note that such a designation customarily includes a comparison with closely associated (similar) taxa; in this case, as previous authors have also noted, *Polyura inopinatus* has no obvious association with any other *Polyura* species. A Type Locality of West New Britain is established. The neotype has been deposited in the Natural History Museum (NHM), London.

## Taxonomy

### Designation of neotype for *inopinatus* Röber, 1940

A neotype of *Charaxes inopinatus* Röber, 1940, is hereby designated. Male (Figs 3, 4), with the following labels: (1) Papua New Guinea, Talasea, West New Britain Province, 600m, 19 Sep, 2015, Chris J. Müller; printed: NEOTYPE / *Charaxes inopinatus* Röber, 1940 / designated by Chris J. Müller & W. John Tennent, March 2018 (NHM, London).

Here we also redescribe *P. inopinatus*, including the male genitalia, since various important diagnostic features were omitted from Röber’s original description. Figs 3–8 depict male specimens of *P. inopinatus*.

Description. ♂ Forewing length 31.5mm (Neotype), Antenna length 14.1mm (Neotype). Head orange-brown; antenna black tipped with orange; thorax and abdomen deep orange-brown on upperside, light orange-brown beneath; legs light-medium brown, with black along the anterior margin of the femur.

Forewing with costa slightly bowed, termen concave and inner margin straight.

Forewing upperside bright orange, a deeper shade in basal area; costa, apex and termen broadly black; discocellulars black, forming a black stripe perpendicular to costa at end of cell; a small postmedian patch of bright orange occupying spaces 5 and 6; a subterminal row of small bright orange spots (of variable shape) in spaces 6–1b, those in the latter space are duplicate; cilia black.

Forewing beneath deep grey-brown with indistinct purple suffusion and chestnut coloured in basal half; three broad (1–2 mm wide) black bars in cell, perpendicular to costa, that in the middle extending into space 2; discocellulars broadly black; the basal and middle cell bars joined with broad diffuse cream borders; a broad cream median area spanning spaces 4 to the inner margin, grading to orange at inner margin, basally thickly (1.5 mm) bordered with black; a small postmedian patch of cream in spaces 5 and 6, also thickly bordered with black basally; an irregular black subterminal band approximately parallel with the termen and widening towards inner margin, bordered narrowly with bluish-white and orange progressively on outer margin at costa and broadly with bright orange at inner margin; cilia brown.

Hindwing with a sharp narrow tail approximately 6mm long at vein 4 and a shorter (2 mm) tail at vein 2; termen scalloped.

Hindwing above bright orange, a slightly deeper shade basally but very pale orange in median area of costa; termen bordered with black (approximately 1mm thick); a row of subterminal black elongated spots, that closest to tornus bifurcated and narrowly dusted basally with purple and white, all subterminal spots basally diffused with black dusting; tornus narrowly blue-white; inner margin broadly orange-brown, white-brown near base; cilia black.

Hindwing beneath with ground colour rich chestnut in basal half, grey-green in distal half; two broad (approximately 1mm wide) black sub-parallel bars in basal and median area, approximately parallel with inner margin and tapering towards tornus, bordered on outer edges with cream (broadest in median area nearest to costa); a postmedian band of crescent-shaped rich red-maroon spots, with purple-white and progressively black basally; a broad (1 mm) black postmedian bar in spaces 1a and 1b, roughly parallel with costa, connecting these spots with inner margin; a row of orange-yellow elongated spots along termen, set in background sky blue terminal area, basally thickly bordered with black and progressively light blue; termen border black (approximately 1mm thick); cilia black.

**Figure 9.**
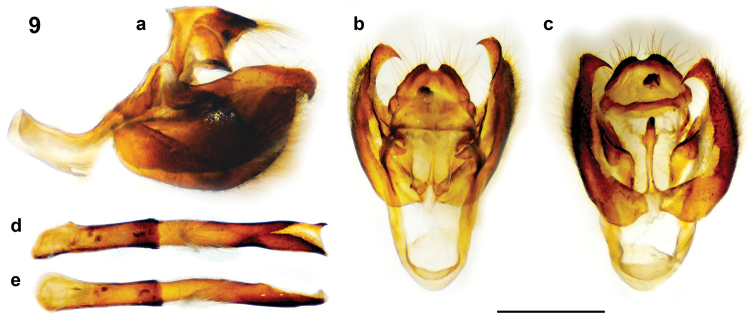
*Polyura inopinatus* male genitalia (Talasea, PNG): **a** genitalia lateral view, aedeagus removed **b** genitalia dorsal view, aedeagus removed **c** genitalia ventral view, aedeagus removed **d** aedeagus lateral view **e** aedeagus dorsal view. Scale bar = 1 mm.

♂ genitalia (Fig. 9). Tegumen elongate (a), oval-shaped dorsally (b) and ventrally (c); sociuncus hooded posteriorly, with prominent dorsal saddle anteriorly at uncus, which is broad dorsally, with fine setae at apex; gnathos brachia short and sharp, plunging downwards; valva broad laterally (a), with pronounced hook at apex; juxta long and narrow, sclerotised apically (c); aedeagus elongate (d, e), with hooked apical spine (d).

### A New Britain connection – and a Sulawesi disconnection?

It seems rather unlikely that such a distinctive species occurs from west of the Moluccas on Sulawesi to the Bismarck Archipelgo, but we believe that Röber’s original specimen also came from New Britain, and not from Sulawesi.

Röber (1940: 104) stated that, unlike most of the material he examined for his Sulawesi checklist, which came from Ludwig Martin, his *inopinatus* came from the stores of Staudinger and Bang-Haas; this was a very large and flourishing business started by Otto Staudinger (1830–1900) in the late 1850s. Andreas Bang-Haas (1846–1925) married Staudinger’s daughter in 1880 and Staudinger and Bang-Haas ran the business together under their joint names until the former’s death in 1900. In 1913 Bang-Haas’ son Otto (1882–1948) took over the business and ran it until his own death. The business was dissolved in September 1948. The Staudinger collection now resides in Berlin; the Bang-Haas commercial material and collections are deposited in Dresden.

Otto Bang-Haas described a number of Lepidoptera species, including two butterflies: *Chilasa moerneri mayrhoferi* and *Delias mayrhoferi* in different issues of the *Entomologische Zeitschrift*, Stuttgart, in 1940. We believe it is significant that both these taxa were described from material collected by “A. Mayrhofer” in the Baining Mountains of East New Britain. These taxa were published without illustrations and, like *P. inopinatus*, were overlooked for many years although, unlike *inopinatus*, type specimens remain extant in the Dresden and Berlin collections. *Delias mayrhoferi* appears to have been completely overlooked – and was redescribed as a new species more than half a century later (*Delias shunichii* Morita, 1996), a synonymy which was resolved by Häuser et al. (2009). For the record, butterfly taxa occurring in East New Britain invariably also occur in West New Britain.

Häuser et al. were unable to establish any biographical data concerning “A. Mayrhofer” (Häuser et al. 2009: 122), and Christoph Häuser (pers comm. to both authors, 2018) has confirmed that this remains the case, although the authors believe Mayrhofer’s given name may have been ‘Alfrons’. The first author carried out research into Mayrhofer and happened across a book by Gail Pool entitled “*Lost Among the Baining: Adventure, Marriage, and Other Fieldwork*” (Pool 2015). In the late sixties, Gail Pool and her husband set off for an adventure in New Guinea. He was a graduate student in anthropology; she an aspiring writer. They met a ‘Father’ Mayrhofer on New Britain and, although there is no direct evidence that this was the Mayrhofer who collected the *Delias* and *Chilasa* that now bear the Mayrhofer name, this may well have been so. Similarly, we have no knowledge that it was Mayrhofer who collected *P. inopinatus* on New Britain – although the period when *Delias mayrhoferi* and *Chilasa moerneri mayrhoferi* were collected (*ca* 1939) and locality (New Britain), and the fact that all passed through the hands of Bang-Haas, raises the distinct possibility that this might have been the case. All three taxa, now known to be from the Bismarck mountains, may well have come from the same locality and source on New Britain.

So Bang-Haas, who provided the *inopinatus* specimen to Röber, had access to material from the mountains of New Britain in exactly the same period. Why he sent the *inopinatus* specimen to Röber is not known: perhaps it was mis-labelled as being from Sulawesi and he knew Röber was working on a checklist; although if he had realised it was an undescribed taxon, he would presumably have retained it and described it himself. On the other hand, Bang-Haas was fundamentally a dealer in butterflies, and may even have sold individuals or a batch of butterflies to Röber, without intimate knowledge of the contents. There is no direct evidence to support such a conclusion, but we believe it is well within the realms of possibility that the solitary *inopinatus* specimen was actually collected by Mayrhofer in the Bainings (or somewhere else) on New Britain, and not on the island of Sulawesi as Röber believed.

The other obvious question now raised is whether there is any direct evidence to suggest that the butterfly was *not* collected on Sulawesi. The short answer is no, but it might be considered relevant that one of the well-known features of butterflies from many families from Sulawesi is a propensity for acutely angled forewings and a distinctly concave forewing outer margin. A good example is that of *Polyura cognatus* Vollenhoven, 1861, which is endemic to Sulawesi and its immediate satellites. *P. inopinatus* does not display this feature. Also, as Vane-Wright and de Jong correctly pointed out (2003: 27), *P. inopinatus* was not encountered during Project Wallace, a year long scientific expedition to the Dumoga Bone National Park on the north-eastern arm of Sulawesi in 1985. The expedition was organised by the Royal Entomological Society and supported by the British Armed Services; various research projects concerning butterflies were undertaken by international scientists. The second author was part of that expedition, spending four months on Sulawesi from May to August 1985, including visiting all the high sub-camps; *P. cognatus* was encountered frequently when it was attracted to sap on trees near the base camp and to baited traps in various localities. It is hard to believe that the distinctive *P. inopinatus* would not have been sighted at all, had it been present.

The Japanese text of Tsukada (1991), which so far as we know has not previously been translated by western researchers, also shows an unsuccessful but concerted effort by Tsukada himself to rediscover the species in its stated locality on Sulawesi: “On April 9, 1991, I still cannot get this rarest species at all. [I] imagine that there are no specimens preserved in the world. It is lost, and nobody can tell where it is. I sent catchers, totally for more than 50 man-days, to the locality to find the butterfly, only in vain. No information was sent back to me from them. A monochrome picture is given by Röber with his original detailed description. I asked Prof Asao Okada to translate the original Deutsch description into Japanese ...”. (Tsukada, 1991: 518 [translation]). The first author visited Lake Tondano (the supposed type locality of *P. inopinatus*) on Sulawesi during 1996 and 2003, specifically in search of *P. inopinatus* but without a glimpse of the insect.

On balance, we believe that the butterfly is not very widespread (*i.e.* from Sulawesi to the Bismarcks) but is instead a Bismarck endemic, wrongly reported to be from Sulawesi when it was described by Röber in 1940.

## Discussion

The enigma of *Polyura inopinatus* has occupied nymphalid specialists and Lepidopterists for decades, due in large part to the fact that the solitary specimen known – the most distinctive representative of the genus – was long believed to have been destroyed during one of the worst international conflicts of our recent history. It may be the case that the actual specimen was seen by very few people – perhaps only Bang-Haas, Röber and whoever took the original monochrome photograph. Entomological research would have been a low priority on the eve of World War II, there would have been no entomological research visitors to Dresden during the war, and by the end of the war the specimen was destroyed. To re-discover the species after more than seven decades, 3,000 kilometres from the published type locality, which was almost certainly erroneous, is as remarkable as it is serendipitous.

Tsukada’s unusual step in presenting a colour illustration of the butterfly from a monochrome photograph and a written description would have been quite a challenge. It is a tribute to the artist, Toshitsugu Endo, that its acceptance (without a translation from the Japanese) as the ‘real thing’, aided by a lack of access to the original journal issued on the eve of World War II, was accomplished so easily. The colours and markings are not perfect, of course; for example, in referring to the underside of the forewings, Röber (1940) said “Posterior wing section wider and diffuse yellowish [translation]”, when the reality is creamy-orange. Also, Röber (1940) declared “The upper side is light reddish-brown”, when the upperside ground colour is bright orange. Another distinctive feature of *inopinatus* is the bright orange distal border to the underside forewing postmedian black band, predictably omitted in Tsukada’s painting since the feature is not mentioned in Röber’s written description. But by and large the painting is fairly accurate. There is some minor variation in the few specimens now seen (cf figs); for example in the line of terminal spots on the upper surface of the hindwing, which may be straight or slightly irregular.

One might question why Tsukada went to the trouble of preparing a colour picture of a butterfly he had never seen and of which a specimen was not available. The answer lies in the Japanese text; he made a concerted effort to obtain the species, and in sending local collectors to what he thought was the type locality, would have needed a colour picture to show them what to look for.

The orange colouration of *P. inopinatus* is like no other *Polyura*, and in this regard it resembles several species of *Charaxes*. The bright orange spots along the subtermen of the underside of the forewing are unlike any other *Polyura*; it is one of the smallest known species of the genus, similar in size to the diminutive *P. athamas* and its siblings from Sundaland. Specimens were observed flying in company with larger numbers of its congener, *P. jupiter* (Butler, 1869), in comparison with which they appeared dwarfed.
